# Potential Energy as an Alternative for Assessing Lower Limb Peak Power in Children: A Bayesian Hierarchical Analysis

**DOI:** 10.3390/ijerph19106300

**Published:** 2022-05-22

**Authors:** Jorge R. Fernandez-Santos, Jose V. Gutierrez-Manzanedo, Pelayo Arroyo-Garcia, Jose Izquierdo-Jurado, Jose L. Gonzalez-Montesinos

**Affiliations:** 1 GALENO Research Group, Department of Physical Education, Faculty of Education Sciences, University of Cádiz, 11519 Cádiz, Spain; 2 Biomedical Research and Innovation Institute of Cádiz (INiBICA) Research Unit, 11009 Cádiz, Spain; 3 Department of Physical Education, Faculty of Education Sciences, University of Cádiz, 11519 Cádiz, Spain; josegu.manzanedo@uca.es (J.V.G.-M.); docmedsport@gmail.com (P.A.-G.); bicijose2004@hotmail.com (J.I.-J.); jgmontesinos@uca.es (J.L.G.-M.)

**Keywords:** children, countermovement jump, potential energy, Bayesian analysis

## Abstract

The aim of this study was to analyze the use of potential energy (PE) as an alternative method to assess peak power of the lower limbs (PP) in children. 815 Spanish children (416 girls; 6–11 years old; Body Mass Index groups (n): underweight = 40, normal weight = 431, overweight = 216, obese = 128) were involved in this study. All participants performed a Countermovement Jump (CMJ) test. PP was calculated using Duncan (PP_DUNCAN_), Gomez-Bruton (PP_GOMEZ_) and PE_CMJ_ formulas. A model with PE_CMJ_ as the predictor variable showed a higher predictive accuracy with PP_DUNCAN_ and PP_GOMEZ_ than CMJ height (R^2^ = 0.99 and 0.97, respectively; ELPD_diff_ = 1037.0 and 646.7, respectively). Moreover, PE_CMJ_ showed a higher linear association with PP_DUNCAN_ and PP_GOMEZ_ across BMI groups than CMJ height (*β*_PECMJ_ range from 0.67 to 0.77 predicting PP_DUNCAN_; and from 0.90 to 1.13 predicting PP_GOMEZ_). Our results provide further support for proposing PE_CMJ_ as an index to measure PP of the lower limbs, taking into account the children’s weight and not only the height of the jump. Therefore, we suggest the use of PE_CMJ_ in physical education classes as a valid method for estimating PP among children when laboratory methods are not feasible.

## 1. Introduction

PP is a measure of great interest in adolescents and children to assess physical fitness, cardiovascular fitness and health status [1,2,3]. For this reason, PP is one of the dimensions of muscular strength commonly included in fitness test batteries for young people, adolescents and children [1,4].

A direct measure of PP is calculated using laboratory tests and a kinetic system (e.g., force platform). However, its use in school settings is limited due to the high cost of the materials used. Field-based fitness tests are a practical alternative to lab tests because they are easy to administer, are relatively safe, involve minimal equipment, and are low in cost; additionally, a large number of participants can be evaluated in a relatively short period of time [5]. Nevertheless, the use of the raw score of a vertical jump in fitness test batteries has two major limitations: (i) an appropriate terminology is not used when denominating “lower body explosive muscular strength” in reference to the capacity to develop strength of the lower limbs. When measuring the raw score of a vertical jump in cm (or m), what is really quantified is the jumping distance as a result of performance; (ii) the body mass of the subject is not taken into account. Therefore, heavier children are penalized when physical education teachers use the vertical jump height as a measure of performance. However, these children mobilize more body mass and probably develop more PP of the lower limbs. In this sense, different authors have reported poor performance of obese subjects in those tests that require propulsion of their own body and explosive strength tests [6,7,8]. To overcome these limitations, and although different researchers have developed formulas to calculate PP from vertical jump test scores and body weight in men, women, youth and children [9,10,11,12,13], our study proposes the use of the potential energy developed by the subject as an alternative for assessing PP of the lower limbs in children.

## 2. Materials and Methods

### 2.1. Study Design

**Figure 1:**
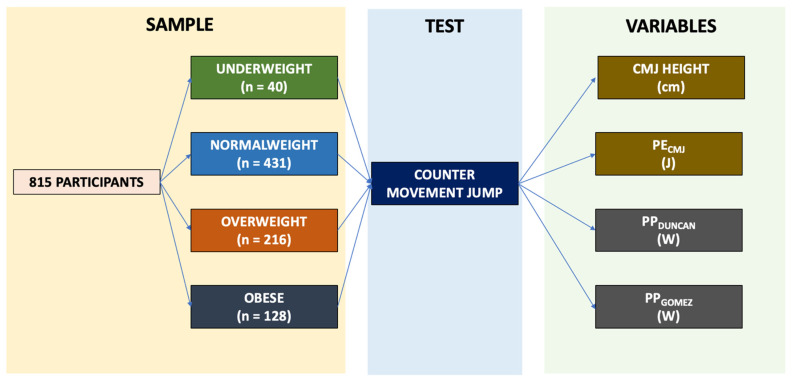
Flowchart of the experiment.

This is a cross-sectional study where all the participants performed a vertical jump test which was used to calculated the using the formulas proposed by Duncan et al. [14] and by Gomez-Bruton et al. [11] (PP_DUNCAN_ and PP_GOMEZ_, respectively). We selected these formulas as the most appropriate since they are the ones that comes closest to the population of this study. Additionally, the potential energy derived from CMJ was also calculated (Section 2.5, Formula (2)). Figure 1 displays a flowchart of the experiment.

### 2.2. Participants

A sample of 815 (416 girls; 6–11 years old; BMI groups (n): underweight = 40, normal-weight = 431, overweight = 216, obese = 128) healthy children participated in the study. All the children were recruited from five elementary schools in the city of Cádiz (Spain). All participants were free of disease and any muscular or skeletal injuries. A comprehensive verbal description of the nature and purpose of the study as well as the experimental risks was given to the children and teachers. Testing sessions were administered at the same time of day and under the same environmental conditions. Participants were asked to avoid any vigorous physical activity for 48 h before the tests. Written informed consent was obtained from parents or legal guardians before the study. This study was approved by the University of Cádiz Doctoral Commission (code: 20090020007122) on 9 October 2009. Moreover, this study was conducted ethically according to the principles of the Declaration of Helsinki II.

### 2.3. Anthropometric Measurements

Height and weight were measured with subjects barefoot and in sports clothes. Weight was measured with an electronic scale (Type SECA 877; range, 0.05 to 200 kg; precision, 0.05 kg). Height was assessed using a stadiometer (Type SECA 213; range 20 to 205 cm; precision, 1 mm). Instruments were calibrated to ensure accurate measures. Body mass index (BMI) was calculated as body mass/height squared (kg/m^2^). Subjects were classified into underweight, normal weight, overweight and obese according to age and sex-specific cut-off points established by Cole et al. [15,16].

### 2.4. Countermovement Jump Test

In an up-right position with legs straight and hands akimbo throughout the test, the participants were asked to jump as high as possible with an early, fast countermovement. The CMJ test has been used to assess lower-body muscular power in children [17]. Before testing sessions, all participants received comprehensive instructions for the tests and completed a 10 min warm-up consisting of jogging and a practice of jumping CMJ to ensure stability in each measure. Each subject completed three CMJs with 45 s of rest between trials. CMJ tests were measured using a SportJump System Pro device (SJS) (DSD, Inc., León, Spain), which is a photocell mat with a photoelectric circuit based on laser beams. It consists of 2 parallel bars, 1 laser transmitter module with 32 laser lights longitudinally placed 3 cm apart, and 1 photosensitive receiver module, with 32 laser receivers placed in front of laser lights. It has a temporal resolution of 0.001 s. This hardware is connected to a laptop where an adaptation of the SportJump-v1.0 software was installed (SportJump-v2.0; DSD Inc.) [18]. The best of three trials was analyzed.

### 2.5. Potential Energy

The potential energy is the energy stored in an object as the result of its vertical position with respect to the ground. This energy is stored as the result of the gravitational attraction of the earth to the object. The higher a subject is elevated, the greater the potential energy. Therefore, there is a direct relation between potential energy and the height of a vertical jump. Additionally, there is a direct relation between gravitational potential energy and the mass of an object. The PE_CMJ_ is reflected in the following equation:

PE_CMJ_ = m · *g* · (*h*_contac_ + *h*_flight_) (1)

where PE_CMJ_ = potential energy derived from CMJ height (J), m = mass of the subject (N), *g* = gravity acceleration 9.79 m·s^−2^ (value *g* in Cadiz, Spain), *h*_contac_ = height of the center of gravity during period of ground contact (m), *h*_flight_ = height of the center of gravity during flight time (m). In this case, since we didn’t know the trajectory of the center of mass of each subject during the time that they are in contact with the ground, we considered the change in potential energy between the instant of take-off and the instant the jumper reached the peak of the jump, calculated by flight time. Therefore, the formula to calculate PE_G_ is reflected in the following equation:

PE_CMJ_ = m · *g* · (*h*_flight_) (2)

where PE = potential energy (J), m = mass of the subject (N), *g* = gravity acceleration 9.79 m·s^−2^ (value *g* in Cadiz, Spain), *h*_flight_ = height of the center of gravity during flight time (m).

### 2.6. Statistical Analysis

Our analysis consisted of modeling the relationship between PP (i.e., PP_DUNCAN_ or PP_GOMEZ_) and the predictors (i.e., CMJ height or PE_CMJ_). Before performing any analysis, both outcomes and predictors were transformed to z-scores using the formula:

(3) $${z-\mathrm{score}}_{i}=(x_{i}-\bar{x})/s$$

where *x_i_* is the value of the variable *x* for the *i*-row, $\bar{x}$ is the mean of the variable *x* and *s* is the sample standard deviation of the variable *x*. A Bayesian multiple regression model was used to analyze the relationship between the outcome and the predictor. This model was defined as follows:
*y_i_*~Normal (*u_i_*, *σ*)[likelihood]*u_i_* = *α* + **β*_1_*Age + **β*_2_*Sex *+ *β*_3_*BMI_GROUP_ + **β*_4_*Predictor + **β*_5_*Predictor: BMI_GROUP_[linear model]*α*~StudentT (0, 2, 3)[prior for intercept]**β*_1_*~Normal (0, 2)[prior for effect of age]**β*_2_*~Normal (0, 2)[prior for effect of sex]**β*_3_*~Normal (0, 2)[prior for effect of BMI_GROUP_]**β*_4_*~Normal (0, 2)[prior for effect of predictor]**β*_5_*~Normal (0, 2)[prior for effect of the interaction predictor: BMI_GROUP_]*σ*~HalfStudentT (0, 2, 3)[prior for residual standard deviation]
where $y_{i}$ is the outcome variable (i.e., PP_DUNCAN_ or PP_GOMEZ_) which was assumed to follow a normal distribution with mean $\mu_{i}$ and residual standard deviation *σ*; the mean $\mu_{i}$ is a linear combination of the parameters *α* (intercept), **β*_1_*, **β*_2_*, **β*_3_*, **β*_4_* and **β*_5_* or effects of the predictor variables age, sex_,_ BMI_GROUP_ and predictor (i.e., CMJ height or PE_CMJ_). Note that Predictor:BMI_GROUP_ denotes the interaction term between the variable predictor (i.e., CMJ or PE_CMJ_) and BMI_GROUP_. Categorical variables were coded using dummy coding (sex: boys = 0, girls = 1; BMI_GROUP_: underweight = 0, normal-weight = 1, overweight = 2, obese = 3). Four different models were fitted with the aforementioned definition:

**Model 1**: Outcome = PP_DUNCAN_/predictor = CMJ height

**Model 2**: Outcome = PP_DUNCAN_/predictor = PE_CMJ_

**Model 3**: Outcome = PP_GOMEZ_/predictor = CMJ height

**Model 4**: Outcome = PP_GOMEZ_/predictor = PE_CMJ_

All hyperparameters of the models were specified individually to follow a weakly informative prior distribution (i.e., a prior that encoded enough information to restrict the plausible range of values of the parameter space but still left a wide range of values to be covered [19]). Once the models were fitted, posterior samples were extracted to calculate individual linear relationships between predictor and outcome across BMI groups.

Three different measures were computed to compare the fitted models. To keep the analysis in line with the scientific literature, a Bayesian version of R^2^ was computed [20]. However, the interpretation is slightly different from the classic R^2^*,* as it should be considered as the proportion of variance explained for new data. The second measure is the leave-one-out information criterion (LOOIC) estimated by leave-one-out cross-validation to assess the expected out-of-sample predictive accuracy of the model [21]. LOOIC is used to calculate the expected log predictive density (ELPD) for a new dataset and to compare the predictive accuracy between models (ELPD_diff_). Models with higher values of ELPD (or lower LOOIC) have better predictive accuracy.

Additionally, the mean of the z-scores by BMI group was plotted for CMJ, EP_CMJ_, PP_DUNCAN_ and PP_GOMEZ_ to visualize similarities among them. Bayesian estimation of the parameters was obtained by using the package brms for the R programming language [22,23]. All parameters estimated showed a good convergence with values of $\hat{R}$ = 1 and number of effective sample size > 1000. Additional information about model definition, prior prediction checking, model convergence and posterior predictive checking can be found in the supplemental file while the code and the dataset to replicate it are stored in https://github.com/JorgeDelro/PEnergy (accessed on 15 May 2022).

## 3. Results

Descriptive characteristics of the sample are displayed in Table 1.

**Figure 2:**
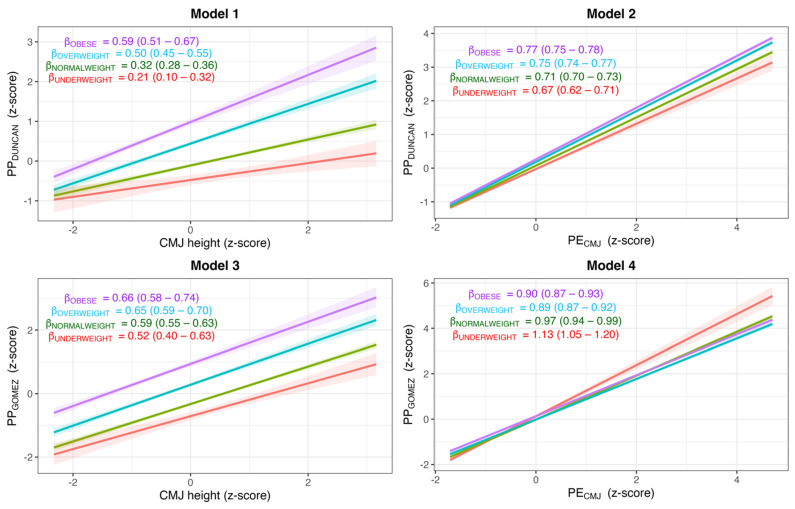
Estimated standardized coefficient (95% credible interval) for each relationship between the outcome (i.e., PP_Duncan_ or PP_Gomez_) and the predictor (i.e., CMJ or PE_CMJ_) by BMI group.

Models 2 and 4 showed a higher predictive accuracy of PP_DUNCAN_ and PP_GOMEZ_ (R^2^ = 0.99 and 0.97, respectively; ELPD_diff_ = 1037.0 and 646.7, respectively), higher linear association of PE_CMJ_ (*β*_PECMJ_ = 0.67 and 1.13, respectively) and lower residual standard deviation (*σ* = 0.05 and 0.14, respectively; *σ*_*β*_ = 0.10 and 0.17, respectively) (Table 2). Additionally, Model 2 and 4 showed a higher linear association of PE_CMJ_ across BMI groups (*β*_PECMJ_ range from 0.67 to 0.77 for Model 2; and from 0.90 to 1.13 for Model 4) (Figure 2).

z-scores of PE_CMJ_ were very similar with the z-scores of PP_DUNCAN_ and PP_GOMEZ_ regardless of the BMI group, in contrast with the z-scores of CMJ height, which followed a different trend across the BMI groups than PE_CMJ_, PP_DUNCAN_ and PP_GOMEZ_ (Figure 2).

## 4. Discussion

The results of this study show that there is a stronger association between PE_CMJ_ with both PP_DUNCAN_ and PP_GOMEZ_ than CMJ height with both PP_DUNCAN_ and PP_GOMEZ_ regardless of the weight status of the subjects.

**Figure 3:**
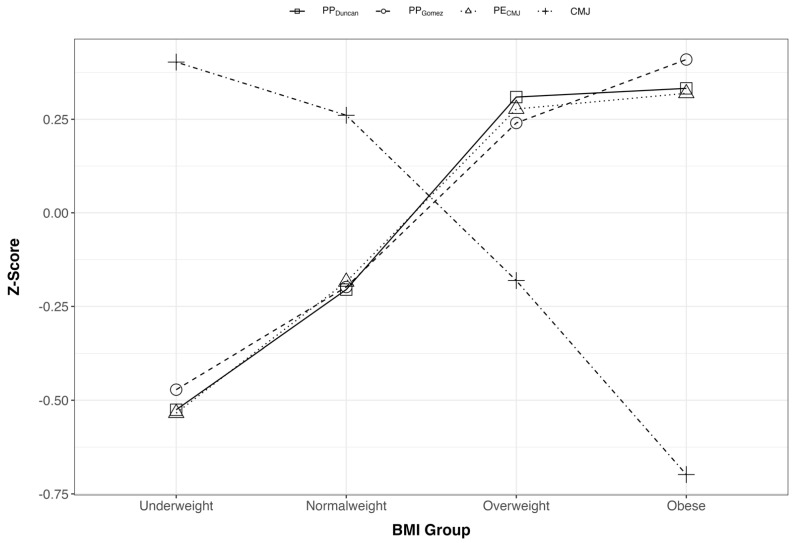
Trend of z-scores values for PP_Duncan_, PP_Gomez_, CMJ, PE_CMJ_ by BMI group.

The results obtained in CMJ test are highly influenced by the BMI of the subject. It can be observed in Figure 3 that to the extent that as BMI group increases, the jump height decrease to a large extent. The differences are minimal in the case of “normal weight” and “underweight” groups, but much more evident if we compare these two groups with “overweight” and “obese” groups. These results agree with the ones obtained in previous studies [4,6,7,8,24].

Nevertheless, if we observe the described trajectory by PP_DUNCAN_, PP_GOMEZ_ and PE_CMJ_ are clearly ascendant with the extent of BMI increase. Moreover, regression analysis shows a higher association of PE_CMJ_ with PP_DUNCAN_ and PP_GOMEZ_ than CMJ height (Table 2 and Figure 2). The results suggest that the height reached in the vertical jump test is not the best indicator of the real force produced by the lower limbs of the subject; so that it would be more appropriate to translate these data to potential energy.

It is clear that if we want to measure developed strength, the displaced mass must be incorporated into the equation, either obtained by means of indirect methods or direct methods such as platform forces [12,14,25,26,27,28]. However, for ease of use in calculation and measurement, it is generally measured by using the height reached by the subject and lower body strength as performance factors, without considering the displaced mass, unaware of the fact that what is measured is not strength not even power, but simply distance or displacement.

The main problems with this method refer to the positions of take-off and landing during a jump, because they are not equal; the angles of the ankle, knee and hip during take-off may have a greater extent than in landing [18,27]. However, Hatze recognizes the usefulness of these methods for the assessment of vertical jump height in most laboratories of biomechanics [29].

For this reason, it is considered necessary to assess PE_CMJ_ developed by the subject when he or she performs a jump, independently of the height reached, which usually is penalized by subject body weight. Therefore, we could consider the possible use of PE_CMJ_ as another method to assess PP.

## 5. Limitations of the Study

Several variables like physical maturation of the participants, physical activity performed and fat-free mass of the lower limbs were not registered in this study. The aforementioned variables could potentially modify the relationship between the outcome and the predictor in the regression analysis. Additionally, the peak power developed in a vertical jump is usually obtained directly using a force platform in laboratory settings. However, peak power was calculated in this study by using two different formulas (PP_DUNCAN_ and PP_GOMEZ_), so that these variables were estimated with additional error.

## 6. Conclusions

This study indicates that PE_CMJ_ is a fair index for knowing which children have more capacity to generate PP of the lower limbs, taking into account the weight and not only the height of the jump. The results of our study suggest that of two subjects that have the same vertical jump height, undoubtedly, the heaviest subject will use greater effort and PE_CMJ_. Physical education teachers can use PE_CMJ_ as an easy method for performing and calculating valid measurements of PP among children when laboratory methods are not feasible. In addition, it will cause greater motivation in these overweight and obese pupils to know that when jumping for the same or even lower heights than other pupils, they will have better results.

## Figures and Tables

**Table 1:** Descriptive characteristics of the sample.

Variables		All	Underweight	Normal-Weight	Overweight	Obese
Sex (n (%))	Boys	399 (49)	16 (40)	212 (49)	110 (51)	61 (48)
	Girls	416 (51)	24 (60)	219 (51)	106 (49)	67 (52)
Age (years)		8.6 ± 1.7	8.8 ± 1.6	8.6 ± 1.7	8.9 ± 16	8.4 ± 15
Height (cm)		136.7 ± 11.8	136.2 ± 11.3	135.0 ± 11.7	139.5 ± 11.7	138.1 ± 11.3
Weight (kg)		35.9 ± 11.1	25.5 ± 4.6	30. 7 ± 6.9	41.0 ± 9.5	48.2 ± 12.0
BMI (kg/m^2^)		18.9 ± 3.7	13.6 ± 0.7	16.6 ± 1.4	20.7 ± 1.8	24.8 ± 2.9
CMJ height (cm)		23.8 ± 5.8	26.2 ± 5.8	25.3 ± 5.7	22.8 ± 5.3	19.8 ± 4.5
PE_CMJ_ (J)		8.5 ± 3.3	6.8 ± 2.3	7.9 ± 3.0	9.5 ± 3.5	9.6 ± 3.6
PP_DUNCAN_ (W)		922.7 ± 340.2	743.9 ± 243.4	852.9 ± 307.7	1027.9 ± 356.5	1035.8 ± 359.7
PP_GOMEZ_ (W)		1007.0 ± 489.5	776.1 ± 403.3	909.9 ± 457.6	1124.5 ± 487.1	1207.6 ± 512.2

BMI indicates body mass index; CMJ, the countermovement jump score; PE_CMJ_, potential energy calculated by using the countermovement jump score; CMJ_DUNCAN_, power calculated using Formula (2).

**Table 2:** Bayesian coefficient of determination (R^2^), leave-one-out information criterion (LOOIC), expected log predictive density (ELPD), difference in ELPD between models with the same outcome (ELPD_diff_), intercept (*α*), standardized regression coefficients (*β*) and residual standard deviation (*σ*).

Outcome	PP_DUNCAN_		PP_GOMEZ_	
Predictor	CMJ Height	PE_CMJ_	CMJ Height	PE_CMJ_
Model	1	2	3	4
Model comparison				
R^2^	0.88 (0.87–0.88)	0.99 (0.99–0.99)	0.86 (0.86–0.87)	0.97 (0.97–0.97)
LOOIC	620.9 ± 50.8	−1453.4 ± 94.5	619.9 ± 51.3	−601.5 ± 66.1
ELPD	−310.5 ± 25.4	726.7 ± 47.2	−346.0 ± 25.6	300.7 ± 33.0
ELPD_DIFF_	1037.0 ± 45.9		646.7 ± 35.2	
Parameter estimates				
*α*	−3.88 (−4.05, −3.70)	−1.49 (−1.56, −1.42)	−3.25 (−3.44, −3.07)	−0.19 (−0.30, −0.07)
*β*_AGE_	0.39 (0.38, 0.41)	0.17 (0.16, 0.18)	0.29 (0.28, 0.31)	0.04 (0.03, 0.05)
*β*_GIRLS_	−0.29 (−0.34, −0.24)	−0.26 (−0.28, −0.25)	0.00 (−0.05, 0.05)	0.00 (−0.03, 0.02)
*β*_NORMALWEIGHT_	0.37 (0.25, 0.49)	0.10 (0.06, 0.14)	0.38 (0.26, 0.51)	−0.14 (−0.21, −0.08)
*β*_OVERWEIGHT_	0.92 (0.79, 1.04)	0.21 (0.17, 0.25)	0.99 (0.86, 1.12)	−0.14 (−0.21, −0.07)
*β*_OBESE_	1.46 (1.31, 1.60)	0.29 (0.25, 0.33)	1.65 (1.50, 1.80)	0.00 (−0.07, 0.07)
*β*_PREDICTOR_	0.21 (0.10, 0.32)	0.67 (0.63, 0.72)	0.52 (0.40, 0.63)	1.13 (1.05, 1.20)
*β*_PREDICTOR:NORMALWEIGHT_	0.11 (0.00, 0.23)	0.04 (0.00, 0.09)	0.07 (−0.05, 0.19)	−0.16 (−0.24, −0.09)
*β*_PREDICTOR:OVERWEIGHT_	0.29 (0.17, 0.41)	0.08 (0.04, 0.13)	0.13 (0.00, 0.26)	−0.23 (−0.31, −0.16)
*β*_PREDICTOR:OBESE_	0.38 (0.24, 0.51)	0.09 (0.05, 0.14)	0.14 (0.01, 0.29)	−0.22 (−0.30, −0.15)
*σ*	0.35 (0.33, 0.37)	0.10 (0.09, 0.10)	0.37 (0.35, 0.39)	0.17 (0.16, 0.17)

R^2^ and parameter estimate results are expressed as mean (95% credible interval); LOOIC, ELPD and ELPD_DIFF_ results are expressed as estimate ± standard error. *β*_PREDICTOR_ for models 1 and 3 = *β*_CMJHEIGHT_, and for models 1 and 4 = *β*_PECMJ_. A positive value in the ELPD_diff_ represents a better predictive accuracy for the model with PE_CMJ_ as predictor. CMJ height indicates the countermovement jump score; PE_CMJ_, potential energy calculated by using the countermovement jump score; PP_DUNCAN_ power calculated using the Duncan et al. (2013) formula; PP_GOMEZ_, power calculated using the Gomez et al. (2019) formula.

## Data Availability

The data, reproducible code and figures of this manuscript can be found at https://github.com/JorgeDelro/PEnergy (accessed on 15 May 2022).
